# Kappa-Carrageenan-Based Dual Crosslinkable Bioink for Extrusion Type Bioprinting

**DOI:** 10.3390/polym12102377

**Published:** 2020-10-15

**Authors:** Wonseop Lim, Gyeong Jin Kim, Hyun Woo Kim, Jiyeon Lee, Xiaowei Zhang, Min Gyeong Kang, Jeong Wook Seo, Jae Min Cha, Hyun Jin Park, Min-Young Lee, Su Ryon Shin, Seon Young Shin, Hojae Bae

**Affiliations:** 1 KU Convergence Science and Technology Institute, Department of Stem Cell and Regenerative Biotechnology, Konkuk University, Seoul 05029, Korea; seop322@nate.com (W.L.); lovewldusv@naver.com (J.L.); zhangxiaowei9304@gmail.com (X.Z.); wjddnr9302@naver.com (J.W.S.); tempast606@naver.com (S.Y.S.); 2 Department of Bioindustrial Technologies, College of Animal Bioscience and Technology, Konkuk University, Seoul 05029, Korea; kkg0118@gmail.com (G.J.K.); vq1004pv@naver.com (M.G.K.); 3 Department of Biotechnology, College of Life Science and Biotechnology, Korea University, Anam-Dong, Seongbuk-Gu, Seoul 02841, Korea; mn40120@naver.com (H.W.K.); hjpark@korea.ac.kr (H.J.P.); 4 Department of Mechatronics, College of Engineering, Incheon National University, Incheon 22012, Korea; j.cha@inu.ac.kr; 5 Smart Healthcare Research Institute, Biomedical Engineering Research Center, Samsung Medical Center, 81, Irwon-ro, Gangnam-gu, Seoul 06351, Korea; my86.lee@samsung.com; 6 Division of Engineering in Medicine, Department of Medicine, Harvard Medical School, Brigham and Women’s Hospital, Cambridge, MA 02139, USA; sshin4@bwh.harvard.edu

**Keywords:** kappa-carrageenan, methacrylic anhydride, dual crosslinking hydrogel, 3D bioprinting

## Abstract

Bioink based 3D bioprinting is a promising new technology that enables fabrication of complex tissue structures with living cells. The printability of the bioink depends on the physical properties such as viscosity. However, the high viscosity bioink puts shear stress on the cells and low viscosity bioink cannot maintain complex tissue structure firmly after the printing. In this work, we applied dual crosslinkable bioink using Kappa-carrageenan (κ-CA) to overcome existing shortcomings. κ-CA has properties such as biocompatibility, biodegradability, shear-thinning and ionic gelation but the difficulty of controlling gelation properties makes it unsuitable for application in 3D bioprinting. This problem was solved by synthesizing methacrylated Kappa-carrageenan (MA-κ-CA), which can be dual crosslinked through ionic and UV (Ultraviolet) crosslinking to form hydrogel using NIH-3T3 cells. Through MA substitutions, the rheological properties of the gel could be controlled to reduce the shear stress. Moreover, bioprinting using the cell-laden MA-κ-CA showed cell compatibility with enhanced shape retention capability. The potential to control the physical properties through dual crosslinking of MA-κ-CA hydrogel is expected to be widely applied in 3D bioprinting applications.

## 1. Introduction

The 3D Bioprinting enables fabrication of complex cell laden structures accurately and efficiently through facilitating specific design modifications based on the needs of researchers [1,2]. For this reason, bioprinting has been attracting attention as a promising technology that can contribute to the development in the field of tissue engineering.

Bioink is a material that includes living cells or biomolecules suitable for reproducing complex tissue structures through 3D bioprinting [3]. To qualify as a bioink, candidate material should satisfy several crucial aspects such as biocompatibility, cell viability and appropriate physical properties. The characteristics of bioink are very diverse and complex and as a consequence developing bioinks is the core of 3D bioprinting technology.

Although number of biocompatible materials have been studied as a bioink candidate, few biomaterials (e.g., Alginate and gelatin) have been successfully optimized for 3D bioprinting applications [3,4,5]. For this reason, it is necessary to develop a bioink for 3D bioprinting that achieves characteristics such as printing suitability, mechanical stability, biodegradability, non-toxicity and cell suitability. In particular, we aim to focus on bioinks that can be optimized with decreased shear stress and increased structural strength of a 3D construct after printing.

As a bioink candidate, we chose carrageenan. Carrageenan is a hydrophilic polymer extracted from algae and is used as a gelling agent and emulsifying agent [6]. Among different kinds of carrageenan, κ-CA has one sulfuric acid group in the repeating disaccharide unix [7] and forms strong and rigid gels through two methods [8]; at low temperatures, agglomeration of spiral coils takes place, forming gels or binding to specific cations such as potassium or calcium to form gels [9]. Due to this dual crosslinking property, κ-CA has been studied in various fields including cartilage tissue regeneration and drug delivery systems [10]. In addition, κ-CA has a characteristic of tunable viscosity depending on concentration, temperature, the presence of ions and molecular weight and has properties of high strength, nontoxic and cellular compatibility [11]. As its shear-thinning and thermo-reversible properties are suitable for bioinks, we chose κ-CA as a bioink material for extrusion-based bioprinting.

However, to become 3D bioprinting compatible, one of the major hurdle to overcome is the intrinsic high viscosity of κ-CA at room temperature (≈25 °C) which requires high pressure during the bioprinting process as it may impair the activity of the cell due to strong physical stimulation. Moreover, although the innate ionic crosslinking property of κ-CA is advantageous for printing accurate shape for extrusion-based bioprinting, this can be affected easily by the change in surrounding environment (e.g., temperature). Therefore, further optimization is required.

In this study, we applied methacrylated Kappa-carrageenan (MA-κ-CA) that shows enhanced physical properties through dual crosslinking mechanisms (ionic and UV crosslinking) for coaxial extrusion-based 3D printing system using NIH-3T3 cells [12]. The substitution reaction of methacrylic groups makes it possible to crosslink the hydrogel by UV, so that the decomposition and swelling characteristics of the hydrogel can be tuned according to the substitution reaction rate [13]. Therefore, dual crosslinkable property is expected to provide structural stability for the printed structure [14].

## 2. Materials and Methods 

### 2.1. Materials

Kappa-carrageenan (κ-CA) from marine sources was purchased from Tokyo Chemical Industry Co. (TCI, Tokyo, Japan). Methacrylic anhydride (MA, contains 2000 ppm topanol A as an inhibitor, 94%) and potassium chloride (KCl, suitable for cell culture) were obtained from Sigma-Aldrich (St. Louis, MO, USA). Sodium hydroxide (NaOH) for pH control was purchased from Duksan Pure Chemicals (Gyeonggi-do, Korea). The ultraviolet light source (Omnicure S2000) was obtained from EXFO Photonic Solutions Inc. (Mississauga, ON, Canada). The photo-initiator for photopolymerization, Irgacure 2959 (2-hydroxy-1-(4-(hydroxyethoxy)phenyl)-2-methyl-1-propanone), was purchased from BASF (Ludwigshafen, Germany). High glucose DMEM was purchased from Welgene (Daegu, Korea), FBS was purchased from Gibco (Waltham, MA, USA) and penicillin streptomycin (P/S) was purchased from Sigma-Aldrich (St. Louis, MO, USA). Live/Dead cell double staining kit and DAPI (4′,6-Diamidino-2-phenylindole dihydrochloride) were purchased from Sigma-Aldrich (St. Louis, MO, USA). Alexa fluor 594 was obtained from thermo fisher scientific (Waltham, MA, USA).

### 2.2. Synthesis of Methacrylated Kappa-Carrageenan

MA-κ-CA was synthesized with a low (4%, w/v), medium (8%, w/v) and high (12%, w/v) degree of substitution using MA as described previously [12]. In brief, κ-CA was dissolved in deionized distilled water with a concentration of 3% (w/v) at 80 °C and MA was added into the κ-CA solution to the desired concentration (4, 8 and 12% w/v) at a rate of 1 mL/min and then stirred for 6 h. Ten moles of (10 M) NaOH were added regularly to the reaction solution to maintain the pH of 8. The reaction-terminated solution was then loaded into the 12–14 kDa dialysis tubing (Diameter: 9 mm, Spectrum; New Brunswick, Canada) and dialyzed for 4 days in deionized distilled water with pH 8 at 4 °C to exclude impurities. The dialyzed solution was filtered using a 1.0 μm membrane filter (cellulose ester, Advantec; Dublin, CA, USA). The purified solution was frozen at −80 °C and lyophilized for 7 days to obtain MA-κ-CA.

### 2.3. ^1^H NMR (Nuclear Magnetic Resonance) Spectroscopy

^1^H NMR (Proton nuclear magnetic resonance) spectroscopy was performed to confirm the chemical structure of the newly synthesized MA-κ-CA. A Varian INOVA 500 MHz FT-NMR (Fourier transform nuclear magnetic resonance) spectrophotometer was used for the comparison and analysis was undertaken of ^1^H NMR spectra of κ-CA and MA-κ-CA with different degrees of substitution ratio (4, 8 and 12%). The analysis was carried out using deuterated water as a solvent for κ-CA and for each MA-κ-CA at 25 °C.

### 2.4. Viscoelastic Properties of MA-κ-CA Prepolymer

Rheological characterization was conducted using a Paar MCR 302 controlled-stress rheometer (Anton Paar, Graz, Austria). κ-CA and MA-κ-CA prepolymer (4, 8 and 12% degree of substitution) samples were prepared at a concentration of 3% (w/v). To analyze the viscoelastic properties of the samples, oscillatory measurements were performed using a parallel plate system with a diameter of 50 mm (PP50, Anton Paar, Graz, Austria) and gap of 1 mm at 25 °C. The linear viscoelastic (LVE) interval between the shear strain and stress was obtained at 10 rad/s by strain sweep tests and a strain value of 0.1% was determined within the LVE range. The viscoelastic values (η*, G′ and G″) was recorded in the angular frequency sweep from 1 to 100 rad/s using Rheocompass software (Anton Paar, Graz, Austria).

### 2.5. Temperature-Sweep Dynamic Shear Profile of MA-κ-CA Prepolymer

To confirm the variation of the dynamic viscoelasticity of MA-κ-CA prepolymer, a temperature sweep test was conducted using a PPTD200 temperature-controlled device (56/I/AIR, Anton Paar, Graz, Austria). The samples were loaded onto the PP50 parallel plate (Anton Paar, Graz, Austria) with a diameter of 50 mm and a gap of 1 mm. The magnitudes of G′, G″ and η* values were observed in the range of 20–75 °C at a heating rate of 3 K/min using a frequency of 0.1 rad/s. Before the measurement, the exposed surface around the parallel plate was coated with corn oil to prevent the loaded samples from drying during the measurements taken at high temperatures.

### 2.6. Preparation of MA-κ-CA Bioink

Since κ-CA has an ionic crosslinking property by bonding with cations, deionized distilled water was used as solvent for bioink preparation. The photo-initiator (Irgacure 2959, BASF, Ludwigshafen, Germany) was completely dissolved in deionized water at 80 °C in a concentration of 0.25% (w/v) and then MA-κ-CA (3%) was dissolved at 50 °C to prepare the bioink.

### 2.7. 3D Fiber Scaffold Printing of MA-κ-CA Bioink

The MA-κ-CA bioink was polymerized by dual crosslinking during the printing process. The crosslinking of κ-CA by cations was facilitated by means of a coaxial nozzle system with internal and external needle sizes of 25G and 18G, respectively. In the printing process, a 3% (w/v) MA-κ-CA bioink was extruded from the internal needle and gelled immediately after contact with the KCl solution flowing via the external needle at the top of the nozzle to form a structure. Latticed constructs (15 mm × 15 mm) were printed using a microfluidic syringe pump with a flow rate of 15 μL/min and printing bed with a moving speed of 280 mm/min at room temperature (25 °C). Immediately after printing, the 3D fiber-deposited scaffolds of MA-κ-CA hydrogel were obtained by irradiating UV (250–450 nm) with 7.3 mW/cm^2^ intensity from a height of 7 cm for 15 s.

### 2.8. Cell Culture and 3D Encapsulation

The NIH-3T3 cell line was obtained from the KCBL (Korean Cell Line Bank; Seoul, Korea) and cultured at 37 °C using high glucose DMEM containing 10% FBS and 1% P/S. For 3D cell encapsulation studies, NIH-3T3 cells were suspended in a prepolymer solution containing 3% MA-κ-CA substituted with 12% methacrylic group and photo-initiator at a final cell concentration of 2 × 10^6^ cells/mL. The mixture was then exposed to 7.3–7.4 mW/cm^2^ UV light (360–380 nm) for 10 s and washed 2 times using warm culture medium before culturing in DMEM in a 37 °C. 3D encapsulation test was performed with a cylindrical shape hydrogel (5 mm diameter and 0.3 mm thickness) during 9 days and extrusion 3D printing with 2% alginate after 5 days.

### 2.9. Dynamic Mechanical Analysis of MA-κ-CA Bioink

To analyze the rheological properties of the MA-κ-CA bioink in relation to the dual crosslinking mechanism, MA-κ-CA bioink was continuously polymerized by UV irradiation after ionic crosslinking. Firstly, each sample was loaded on a measuring bed and 1 mL of 500 mM KCl (potassium chloride) solution was pipetted around the sample to induce ionic crosslinking. Then, measurements were carried out with a frequency of 1 rad/s at 0.1% strain after 10 s from KCl dispensing. After the ionic crosslinking step (30 min), the additional variation of dynamic mechanical properties caused by photopolymerization was observed by irradiation of the sample with UV of 7.3 mW/cm^2^ intensity. Dynamic properties of MA-κ-CA bioink were assessed in real time using stainless steel parallel probe (PP08, Anton Paar, Graz, Austria) with a 1.5 mm gap maintained at 25 °C and G′, G″ were recorded.

### 2.10. Morphology of Bioprinted MA-κ-CA Fiber Scaffolds

The cross-sectional morphology of bioprinted MA-κ-CA fiber scaffolds was observed using a Hitachi model S-4300 (Tokyo, Japan) Field Emission-Scanning Electron Microscope (FE-SEM). To retain the morphology of the samples, the MA-κ-CA fiber scaffold was irradiated with UV for 15 s followed by lyophilization. The cross-sectional MA-κ-CA scaffold was fixed on carbon tape and coated with platinum using a Hitachi E-1030 sputter coater (Tokyo, Japan) for 3 min.

### 2.11. Mechanical Analysis

To perform mechanical analysis, the MA-κ-CA bioink based test specimens were fabricated as follows. First, the prepolymer solution was pipetted into the round shaped PDMS (polydimethylsiloxane) mold 8 mm in diameter and 2 mm in thickness. A PDMS mold was then put on an agarose gel (500 mM KCl and 1.5% agarose) for 30 min incubation to allow the potassium ion (K^+^) to fully diffuse for MA-κ-CA gelation. Then, the MA-κ-CA hydrogel was exposed to 7.3–7.4 mW/cm^2^ UV light (360–380 nm) for 100 s. After washing with DPBS 2–3 times, the hydrogel was incubated in DPBS at 37 °C for 24 h. Finally, crosslinked hydrogel samples were subject to a Young’s modulus test using a CT3 Texture Analyzer (Brookfield Engineering Laboratory, Stoughton, MA, USA). For the analysis, a 5 to 15% strain was selected to determine the Young’s modulus as the slope of the linear region.

### 2.12. Swelling Test

For the swelling test, the MA-κ-CA hydrogel specimens were prepared as described in the previous section (mechanical analysis). For the analysis, the MA-κ-CA hydrogel was put into a 1.5 mL Eppendorf tube and incubated in 1 mL 1X DPBS (Dulbecco’s phosphate-buffered saline) at 37 °C for 24 h. Then, the hydrogel sample was removed from DPBS and washed 2–3 times using distilled water. The swollen weight was recorded after removing excess water and then the sample was lyophilized and the mass of dry weight was recorded. The mass swelling ratio was calculated using the ratio of swollen hydrogel mass to the dried polymer.

### 2.13. Degradation Test

The prepared MA-*κ*-CA hydrogel (cylindrical shape, 8 mm diameter and 2 mm thickness) was put into a 1.5 mL Eppendorf tube and incubated in 1 mL DPBS and high glucose DMEM (Dulbecco’s Modified Eagle Medium) with 10% FBS (fetal bovine serum) at 37 °C for 24 h and the MA-*κ*-CA hydrogel was washed 2–3 times with distilled water. Then, the initial hydrogel and degraded hydrogel were lyophilized and mass of the dried initial and degraded hydrogel weight was recorded. The weight loss was calculated by dividing dry mass of initial hydrogel by degraded hydrogel.

## 3. Results and Discussion

### 3.1. Synthesis of Methacrylated Kappa-Carrageenan

The shear-thinning and ionic crosslinking property of κ-CA is suitable as a bioink for extrusion-based bioprinting systems. However, the κ-CA hydrogel formed solely by ionic bonding may become unstable under physiological condition as frequent ion concentration changes may take place in extracellular spaces [15]. To compensate for this limitation, the substitution of the hydroxyl group (-OH) of κ-CA with methacrylic group of MA enables photopolymerization, thereby enhancing the stability of the molecular structure [12]. The synthesized MA-κ-CA thus exhibits biphasic properties including both the ionic bond of κ-CA and the UV photopolymerization properties of substituted methacrylic groups.

**Figure 1:**
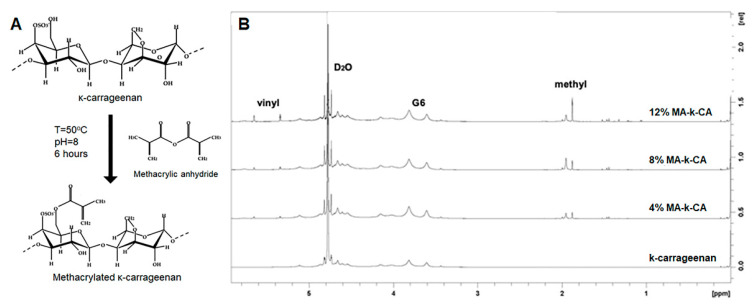
Schematic representation of the synthesis of MA-κ-CA and ^1^H Nuclear Magnetic Resonance (NMR) spectra of κ-CA and MA-κ-CA (4, 8 and 12%). (**A**) Chemical modification of MA-κ-CA and (**B**) ^1^H NMR spectra of κ-CA and MA-κ-CA recorded in deuterium oxide (D_2_O). The vinyl group peak was located at chemical shift (δ) = 5.3–5.8 ppm and methyl group peak at δ = 1.8–2 ppm. The D_2_O peak was located at δ = 4.6–4.8 ppm and β-galactose subunit of κ-CA (G6) were found around δ = 3.5–3.9 ppm.

The MA-κ-CA resulted in a different MA substitution ratio depending on the concentration of MA added in the synthesis process. The ^1^H NMR spectrum of MA-κ-CA according to MA substitution is shown in Figure 1. The vinyl group peak (δ = 5.3–5.8 ppm) and the methyl group peak (δ = 1.8–2 ppm) by substitution of the methacrylic group was observed in the NMR spectra of the MA-κ-CA conjugate against κ-CA. The MA substitution ratios of the MA-κ-CA were 4%, 8% and 12%, respectively.

### 3.2. Rheological Properties of MA-κ-CA Prepolymer

#### 3.2.1. Viscoelastic Properties

**Figure 2:**
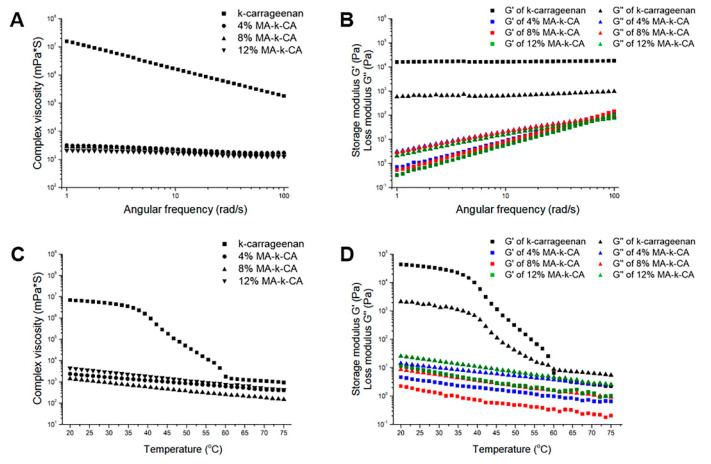
Angular frequency and temperature dependent rheological properties. Rheological properties of the κ-CA and MA-κ-CA (4, 8 and 12%) prepolymer solution at printing temperature (25 °C). (**A**) Complex viscosity (**B**) Storage modulus (G′) and Loss modulus (G″). Temperature dependent rheological properties of the κ-CA and 4, 8 and 12% MA-κ-CA prepolymer. (**C**) Complex viscosity (**D**) Storage modulus (G′) and loss modulus (G″).

It has been reported that the κ-CA prepolymer has a shear-thinning property with decreasing viscosity as frequency increases [16]. As shown in Figure 2A, the viscosity (1.58 × 10^7^ mPa·s) of κ-CA at the low frequency (1 rad/s) was significantly higher than the viscosity (1.81 × 10^5^ mPa·s) at high frequency (100 rad/s). But, the viscosity at high frequency is still high to be suitable for 3D bioprinting. These shear-thinning behaviors were similarly observed in the MA-κ-CA prepolymer solutions (4, 8 and 12% degree of substitution). Correspondingly, the overall shear-thinning property was significantly reduced for MA-κ-CA solutions making it suitable for 3D bioprinting. The viscosity (4%; 3.18 × 10^4^ mPa·s, 8%; 2.83 × 10^4^ mPa·s, 12%; 2.11 × 10^4^ mPa·s) of MA-κ-CA at low frequency (1 rad/s) was higher than the viscosity (4%; 1.72 × 10^4^ mPa·s, 8%; 1.71 × 10^4^ mPa·s, 12%; 1.28 × 10^4^ mPa·s) at high frequency (100 rad/s). In addition, as the degree of substitution of κ-CA increased, the magnitude of viscosity decreased. Correspondingly, it can be inferred that the entangled double-helix structure of κ-CA has been disrupted.

In addition, the values of Storage modulus (G′) and Loss modulus (G″) for κ-CA and MA-κ-CA prepolymer with 4%, 8% and 12% degree of substitution with increasing frequency were observed (Figure 2B). κ-CA exhibited gel-like properties with G′ values higher than G″ values in the measured frequency range, whereas MA-κ-CA prepolymers showed liquid-like profiles in the range below 60 rad/s which is applicable to the bioprinting process by reducing the stress on bioink.

#### 3.2.2. Temperature-Sweep Dynamic Shear Profile

κ-CA prepolymer has been reported to exhibit temperature sensitive reversible sol-gel transition in the absence of salt [17]. At low temperatures, single-stranded polymer chains are entangled in a double helical structure to form a stable gel. As temperature increases, the viscosity decreases markedly due to the increase in thermodynamic instability of the κ-CA polymer chains, indicating the sol behavior. The high viscosity of κ-CA at low temperatures, depending on the nature of the sol-gel transition, represents a limitation of printing performance. In particular since bioprinting often proceeds at room temperature, it is important to confirm the rheological characteristics of the bioink at the corresponding temperature.

The viscoelasticity (G′; storage modulus, G″; loss modulus and η*; complex viscosity) of κ-CA and MA-κ-CA prepolymer according to the temperature sweep [18,19]. As shown in Figure 2C,D, the complex viscosity of κ-CA at room temperature (25 °C) was measured at 6.01 × 10^6^ mPa·s, which was significantly higher than those values (ranged from 6.33 × 10^3^ mPa·s for 4% MA-κ-CA to 2.57 × 10^3^ mPa·s for 12% MA-κ-CA) of MA-κ-CA prepolymer (Figure 2C). In addition, a remarkable loss in viscosity of κ-CA was observed as the temperature increased from 37 °C to 60 °C and a decrease in viscosity to a level similar to the MA-κ-CA prepolymer range was exhibited when heated further. In particular at approximately 60 °C, a crossover of G′ and G″ was observed, suggesting reversible phase change from gel to sol (Figure 2D). On the other hand, for MA-κ-CA prepolymer, it was confirmed that the crossover of G′ and G″ that occurred with the temperature increase was eliminated by MA substitution. Furthermore, it can be confirmed that the higher the substitution ratio of the methacrylic group residues to the hydroxyl group, lower viscosity was observed at the same temperature due to the reduced entanglement of the double helix polymer chains. This property of the MA-κ-CA prepolymer can be useful in bioink applications as it is related to higher survival rate of cells by reducing the external force on the cells during the printing process at room temperature (or lower).

### 3.3. Characterization of MA-κ-CA Hydrogel

#### 3.3.1. Dynamic Rheological Properties

**Figure 3:**
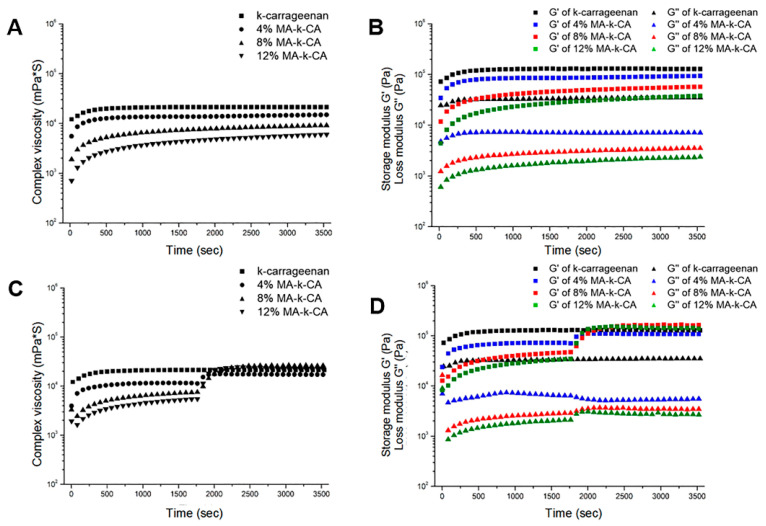
Ionic and UV crosslinking dependent rheological properties. Rheological properties of κ-CA and 4, 8 and 12% MA-κ-CA hydrogel during ionic crosslinking for 1 h (3600 s) (**A**) Complex viscosity (**B**) Storage modulus (G′) and Loss modulus (G″). Rheological properties of κ-CA and 4%, 8%, 12% MA-κ-CA hydrogel during ionic and UV crosslinking for 1 h (3600 s) (**C**) Complex viscosity (**D**) Storage modulus (G′) and Loss modulus (G″).

The viscoelasticity of κ-CA and MA-κ-CA hydrogel was measured for 1 h starting from 10 s after the KCl solution was dispensed. The 4, 8 and 12% MA-κ-CA prepolymer showed a liquid-like profile in which the G″ value was higher than the G′ value, whereas gel-like properties (G′ > G″) were observed in MA-κ-CA hydrogel. This indicates that the MA-κ-CA bioink reacts immediately with KCl to form a gel. When the MA substitution ratio of the MA-κ-CA hydrogel is high, the scope for ionic bond formation by the sulfate group is lowered. Therefore, a higher MA substitution ratio leads to a lower ratio of physical gelation by ionic bonds and the decrease of the G′ and G″ values was observed as the MA substitution ratio increased (Figure 3A,B). This means that a high MA substitution ratio leads to low physical gelation by ionic bonds and as such a decrease in viscoelastic values (η*, G′ and G″) was observed as the MA replacement ratio increased. Consequently, the higher the MA substitution ratio, the stronger the covalent bond through photopolymerization is induced due to methacrylic groups.

Viscoelastic values were plotted during constant ion exposure and UV irradiation for 30 min (Figure 3C,D). When the ion-crosslinked hydrogel was irradiated with UV light, the G′ values of the MA-κ-CA hydrogel increased. In particular, G′ values of 8 and 12% MA-κ-CA hydrogel were higher than G′ values of κ-CA (Figure 3C). Viscoelasticity was calculated to obtain loss angle by dividing G″ by G′ as the ratio of elastic and viscos component. Plots of κ-CA and MA-κ-CA hydrogel reached equilibrium within 1800 s (30 min) after ionic crosslinking and showed higher loss angle values with a decreased substitution ratio of MA (Figure 3D). 

#### 3.3.2. Morphology of MA-κ-CA Fiber Scaffolds 

**Figure 4:**
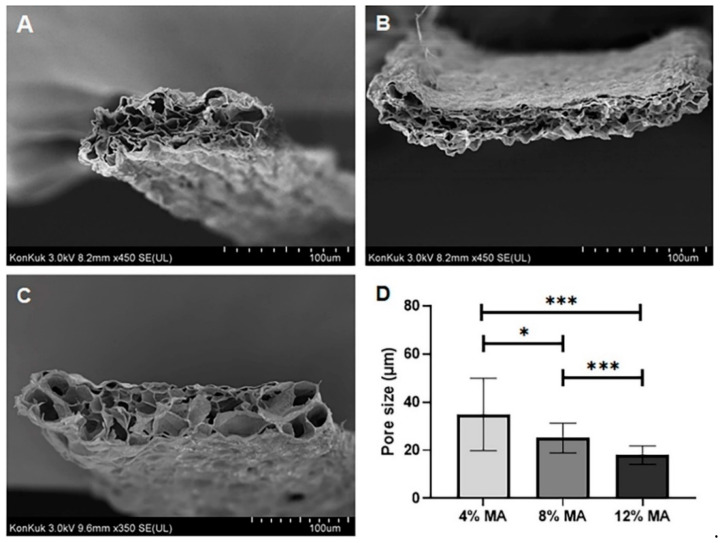
Scanning electron microscopy (SEM) images of MA-κ-CA hydrogel fiber (cross-sectional view). SEM images of cross-sectioned area of lyophilized MA-κ-CA fiber hydrogel formed by ionic and UV crosslinking (**A**) 4% MA-κ-CA, (**B**) 8% MA-κ-CA, (**C**) 12% MA-κ-CA. (**D**) Pore size according to substitution ratio of MA (* *p* < 0.05, *** *p* < 0.001).

To confirm the morphology of MA-κ-CA hydrogel after the dual crosslinking process according to the MA substitution ratio, cross-sections of lyophilized samples after dual crosslinking were observed using a Field Emission Scanning Electron Microscope (FE-SEM) (Figure 4). As shown in Figure 4, the cross-sectional morphology of the 4, 8 and 12% MA-κ-CA hydrogel showed a porous fiber scaffold. As the substitution ratio increased, the overall pore size decreased. The porous 3D structure of the hydrogel not only provides space for the cells to reside in the hydrogel but also provides a microenvironment that can supply nutrients and oxygen to the cells [20].

#### 3.3.3. Mechanical Properties

**Figure 5:**
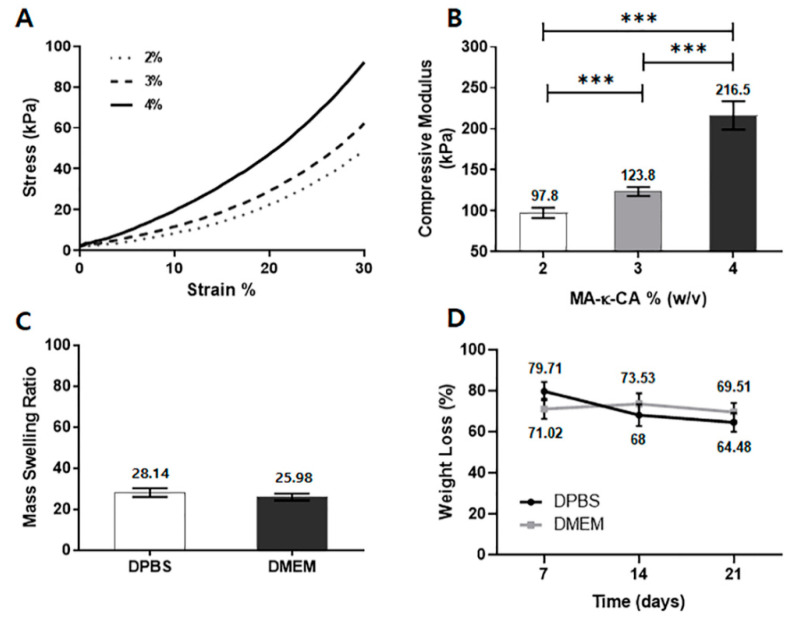
Mechanical properties, mass swelling ratio and degradation behavior of MA-κ-CA hydrogel. (**A**) Representative curves of 2%, 3% and 4% MA-κ-CA at 12% degree of methacrylation. (**B**) Young’s modulus of 2%, 3% and 4% (w/v) MA-κ-CA at 12% degree of methacrylation. (**C**) The mass swelling ratio of 3% (w/v) MA-κ-CA hydrogels in DPBS and DMEM. (**D**) Representation of weight loss percentage according to time of 3% MA-κ-CA hydrogels, immersed in DPBS and DMEM at 37 °C. (*n* = 3) ± standard deviation (*** *p* < 0.001).

The mechanical properties of the hydrogel are crucial to material design as they affect cell function and differentiation [21,22]. To confirm the effect of the MA-κ-CA hydrogel concentration on the mechanical properties, we chose the highest substitution ratio to better represent the mechanical properties. The concentration of the MA-κ-CA hydrogel (8 mm diameter and 2 mm thickness cylindrical shaped) substituted with 12% methacrylic group was set to 2%, 3% and 4% (w/v). As the concentration of MA-κ-CA hydrogel increased (2%, 3% and 4%), the stiffness increased at all strain levels (Figure 5A). Young’s modulus also increased as concentration of MA-κ-CA hydrogel increased. For MA-κ-CA hydrogel concentrations of 2%, 3% and 4%, the compressive strengths of the resultant hydrogel were 97.8, 123.8 and 216.5 kPa, respectively (Figure 5B). 

#### 3.3.4. Swelling and Degradation Behavior

The swelling properties have a significant effect on the physical properties of the hydrogel and the fidelity of the desired micropattern [13]. The degree of swelling depends on the type of polymer, solvent, network size and their interactions [23]. MA-κ-CA hydrogel (3% w/v) swelling test was performed for 24 h at 37 °C in DPBS (ions content) and DMEM (with FBS; ions and protein content). In the swelling test, the extent of the swelling ratio was slightly higher in DPBS than in DMEM (Figure 5C). This may be due to ions in DPBS which destabilizes the crosslinking network by substitution of the ions present in the network of MA-κ-CA hydrogel. 

Further, the degradation test was performed in DPBS and DMEM to assess the stability. It was measured every 7 days. During the first 7 days, the weight loss of MA-κ-CA hydrogel in DPBS gradually decreased 79.71%. After the measurement period, the overall weight steadily decreased to 68% at 14 days and 69.51 at 21 days. In the case of MA-κ-CA hydrogel in DMEM, the weight loss decreased to 71.02% at first 7 days. But, after the 7 days, the weight remained constant throughout the degradation test period; 73.53% at 14 days and 69.51% at 21 days (Figure 5D).

#### 3.3.5. 3D Encapsulation in MA-κ-CA Hydrogel

**Figure 6:**
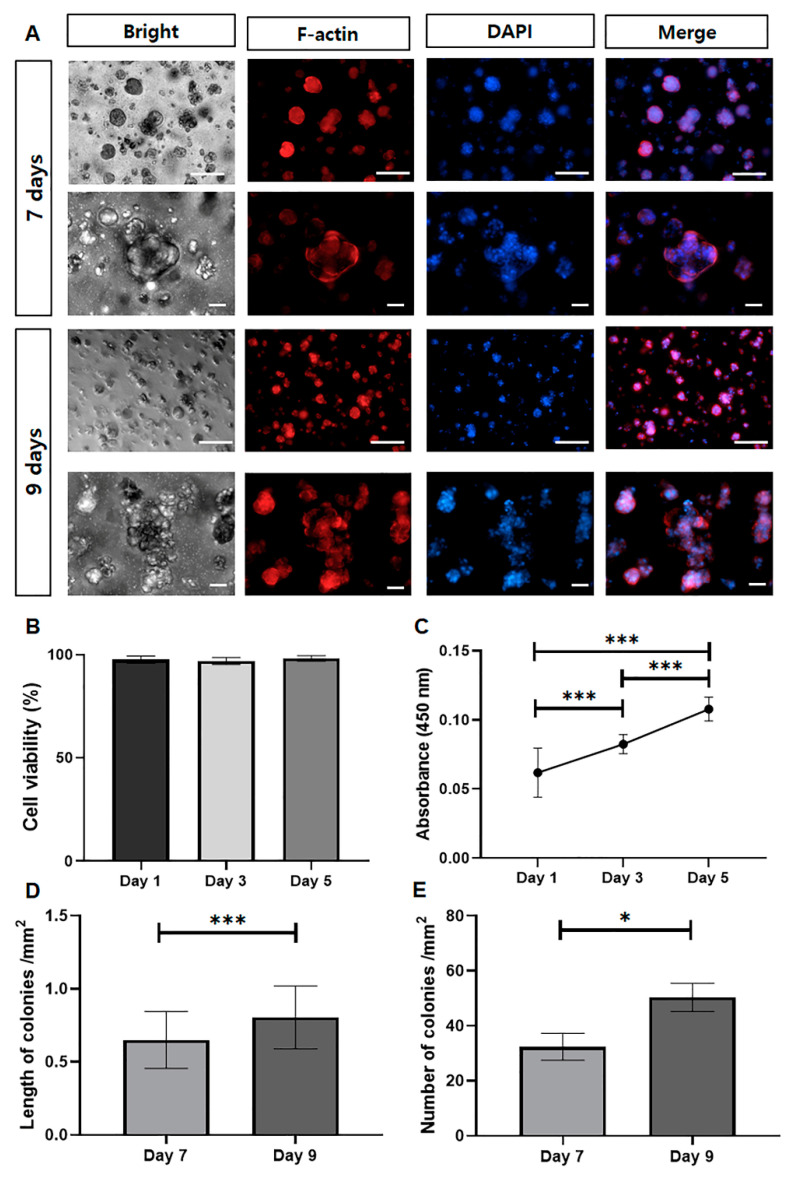
3D encapsulation in MA-κ-CA hydrogel assay. (**A**) DAPI/F-actin staining image at day 7 and 9. (Scale bar = x100; 200 μm, x200; 100 μm). (**B**) Cell viability assay and (**C**) CCK8 assay at day 1, 3 and 5. (**D**) Length of colonies and (**E**) Number of colonies at day 7 and 9 (* *p* < 0.05, *** *p* < 0.001).

3D encapsulation test is essential for measuring cell behavior in 3D tissue-like environment [24]. To measure the cell behavior (cell viability and adhesion), encapsulated NIH-3T3 (fibroblast derived from mouse) cells were cultured in MA-κ-CA hydrogel for 9 days. NIH-3T3 fibroblasts were employed since NIH-3T3 fibroblasts are commonly used in experiments as a control with the capability of forming ECMs such as collagen fiber and glycosaminoglycan and so forth. Also, NIH-3T3 cells has characteristics that are particularly good for cellular differentiation and proliferation within Extracellular matrix. As shown in Figure 6A, DAPI/F-actin staining was used to investigate cell morphology of encapsulated NIH-3T3 cells and it could be observed that NIH-3T3 fibroblasts forming cell aggregates in MA-κ-CA hydrogel for 9 days. DAPI staining was used for nuclear staining and F-actin staining was used for cytoskeletal actin filaments staining. Cell viability assay was used to calculate the ratio of live/total (live and dead) cell (Figure 6B) and CCK-8 (Figure 6C). Encapsulated NIH-3T3 cells showed high cell viability during 5 days of culture period and demonstrated that 3D encapsulated cells in MA-κ-CA hydrogel can survive long-term period. To analyze the colonized cell aggregates, two measurements were performed: Length of colonies (Figure 6D) and the number of colonies (Figure 6E). According to calculation, it was confirmed that length of colonies, the spheroid area and number of colonies increased. These results demonstrated that MA-κ-CA hydrogel are useful in a range of 3D cell culture long-term period with high cell viability. 

### 3.4. 3D Bioprinting Using MA-κ-CA Bioink

**Figure 7:**
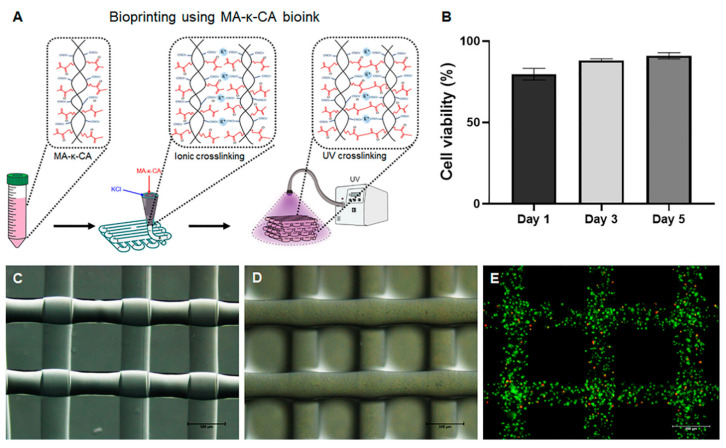
Dual crosslinking (Ionic and UV) mechanism and microscopic images of printed construct. (**A**) Ionic and UV crosslinking mechanism of cell-laden MA-κ-CA bioink for 3D bioprinting. (**B**) Cell viability assay of 3D printed cell-laden MA-κ-CA hydrogel. (**C**) Microscopic image of MA-κ-CA hydrogel fiber. (**D**) Microscopic image of encapsulated MA-κ-CA hydrogel fiber at 24 h. (**E**) Live/Dead assay image of 3D printed MA-κ-CA hydrogel fiber with 2% alginate at day 5 (Scale bars = 500 μm).

κ-CA is capable of biding specific cations (K^+^ and Ca^2+^, etc.) to form gels [9]. In particular, potassium ions (K^+^) efficiently combine with sulfuric acid groups (H_2_SO_4_) of κ-CA to form gels with high elasticity [25]. The ionic crosslinking of κ-CA forms a gel by condensing a 3D network through physical interactions between double helix structures. MA-κ-CA can be crosslinked through photopolymerization by UV. In addition, although MA substitution lowers the ratio of the ionic bond formation by the sulfate group, MA-κ-CA can maintain physical crosslinking properties through ionic bonding. The sulfated and methacrylic groups present in MA-κ-CA have independent crosslinking mechanisms and can modulate the physical properties and cellular compatibility of MA-κ-CA hydrogels according to the substitution ratio of MA (Figure 7).

In this study, our main objective was to apply dual crosslinkable MA-κ-CA as a bioink using coaxial extrusion based bioprinting methods. Consequently, MA-κ-CA bioink was extruded through a coaxial inner needle and at the same time KCl solution was extruded on the outer needle to instantly crosslink in order to maintain the morphology of the multilayer fiber structure. Through this step, it was verified that the physical crosslinking step of MA-κ-CA by ion-binding provided a sufficient amount of bonding to maintain the structure by crosslinking with cations immediately after printing, despite the low viscosity of MA-κ-CA.

After the initial printing step, the methacrylic groups of MA-κ-CA are exposed to UV for photopolymerization (Figure 7A). Compared to ionic crosslinking, UV crosslinked MA-κ-CA hydrogel resulted in latticed constructs with high mechanical strength. Physical crosslinking by potassium ions forms relatively strong bonds with high brittleness but rapidly hydrolyzes, while covalent crosslinking by UV results in flexible bonds capable of retaining moisture between molecules and maintaining structure. Therefore, the water content and the degree of degradation of the hydrogel formed by the dual crosslinking can be determined by controlling the MA substitution ratio of MA-κ-CA. Thus, the physical properties of the hydrogel, such as swelling, degradation and strength, can be fine-tuned by adjusting the degree of methacrylation of MA-κ-CA and this tunability allows MA-κ-CA based bioink to be applied to tissue engineering field which requires materials with various physical properties [10,26]. The cell viability assay was performed to calculate the ratio of live/total (live and dead) cell at days 1, 3 and 5 (Figure 7B). The MA-κ-CA hydrogel fiber and cell-laden hydrogel fiber were printed in a grid pattern (10 mm in length and 10 mm in width, Figure 7C–E). As shown in Figure 7E, the cell-laden scaffold displayed a 3D arrangement under visualization using Lionheart FX (Biotek, Winooski, VT, USA). The NIH-3T3 cells encapsulated in printed hydrogel showed cell spheroid structure with high cell viability as can be seen in cylindrical shaped hydrogel. These data suggest that fibroblasts encapsulated in 3D printed MA-κ-CA hydrogel has inherent potential to self-organize into 3D aggregated spheroids. Further, 3D printed MA-κ-CA hydrogel could be used to control cellular conditions such as formation of cellular spheroids or elongated cellular networks by mixing with other methacrylated hydrogel or polymer [27].

## 4. Conclusions

Kappa-carrageenan (κ-CA) has been studied in the field of tissue engineering due to its properties such as high strength, biocompatibility and crosslinking mechanism. However, it has the limitation of being difficult to be used due to its high viscosity and therefore control gelling. To solve this problem, we have synthesized MA-κ-CA, a carrageenan polymer substituted with MA. The characteristics of MA-κ-CA satisfied the important requirement as a bioink in that it exhibits shear-thinning behavior, as well as tunable rheological and mechanical properties for bioprinting. Most importantly, MA-κ-CA showed a suitable range of rheological properties as a bioink due to significantly reduced viscosity compared to its natural form (κ-CA) along with enhanced stability against change in temperature. Further, tunability of MA-κ-CA according to degree of substitution of MA provides control over various range of rheological and mechanical properties for printing purposes. In the 3D cell encapsulation, good cell viability and formation of cellular spheroid in MA-κ-CA would be good advantage in 3D printing. As it is made of single polymer, through uncomplicated optimization MA substitution, MA-κ-CA shows a great potential as a bioink candidate that can be usefully applied to bioprinting based tissue engineering applications.
